# “How is social media used for learning?”: relationships between social media use by medical students with their self-regulated learning skills

**DOI:** 10.1186/s12909-024-05222-7

**Published:** 2024-03-05

**Authors:** Ardi Findyartini, Nadia Greviana, Chaina Hanum, Elvan Wiyarta, Justinus Kurniabudhi Novarianto, Yehuda Tri Nugroho Supranoto, Maritza Andreanne Rafa Ayusha, Dwita Oktaria, AASA Santhi Sueningrum, Yuni Susanti Pratiwi, Eti Poncorini Pamungkasari, Gita Sekar Prihanti, Rahma Tsania Zhuhra, Yoanita Widjaja, Diani Puspa Wijaya, Komal Atta

**Affiliations:** 1 Medical Education Center, Department of Medical Education, Faculty of Medicine, Indonesia Medical Education and Research Institute (IMERI), Universitas Indonesia, Jakarta, Indonesia; 2 Department of Medical Education, Faculty of Medicine, Universitas Indonesia, Jakarta, Indonesia; 3 Undergraduate Medical Program, Faculty of Medicine, Universitas Indonesia, Jakarta, Indonesia; 4 Undergraduate Medical Program, Faculty of Medicine, Universitas Jember, Jember, Indonesia; 5 Undergraduate Medical Program, Faculty of Medicine, Universitas Lampung, Bandar Lampung, Indonesia; 6 Department of Biomedical Science, Faculty of Medicine, Universitas Padjadjaran, Bandung, Indonesia; 7 Medical Education Unit, Faculty of Medicine, Universitas Sebelas Maret, Surakarta, Indonesia; 8 Department of Medical Education, Faculty of Medicine, Universitas Muhammadiyah Malang, Malang, Indonesia; 9 Department of Medical Education, Faculty of Medicine, Universitas Andalas, Padang, Indonesia; 10 Medical Education Unit, Faculty of Medicine, Universitas Tarumanagara, Jakarta, Indonesia; 11 Department of Medical Education, Faculty of Medicine, Universitas Islam Indonesia, Yogyakarta, Indonesia; 12 Medical Education Unit, Faculty of Medicine, Soegijapranata Catholic University, Semarang, Indonesia; 13 Department of Medical Education, University Medical & Dental College, University of Faisalabad, Faisalabad, Pakistan

**Keywords:** Social media, Self-regulated learning, Undergraduate medical education

## Abstract

**Background:**

Social media is widely used by medical students, including for learning purposes since it facilitates their involvement in the communities of inquiry where they can share, express, and engage in the development of knowledge. Navigating the use of social media requires self-regulated learning (SRL) skills. Hence, studies on the relationships between social media use and SRL skills are necessary.

**Aim:**

This study aims to investigate the relationships between social media use and students’ SRL skills.

**Methods:**

A cross-sectional study was conducted using two validated questionnaires: the Social Networking Sites for Medical Education questionnaire (SNSME, 19 items) and the Motivated Strategies for Learning Questionnaire (MSLQ, 81 items). Cross-cultural adaptation and exploratory factor analysis (EFA) were also completed for the SNSME questionnaire, followed by descriptive and bivariate analysis.

**Results and discussion:**

The SNSME questionnaire is valid for use in the current setting and consists of three subscales: (1) attitudes towards the use of social media for learning and knowledge development, (2) the use of social media for information sharing and interaction, and (3) the use of social media for knowledge development and research. Among 1,122 respondents, male students presented lower scores than female students in the total score of social media for learning (80 vs. 82, p 0.007), and public medical students showed higher scores in terms of attitudes towards the use of social media for learning and knowledge development compared to private medical students (83 vs. 81, p 0.007). The differences in SRL scores for different education stages and among students from public and private medical schools were statistically significant (426 vs. 418, p 0.003, and 436 vs. 418, *p* < 0.001, respectively). Levels of correlation between social media use and SRL scores were low to moderate (R 0.195–0.462, *p* < 0.001).

**Conclusions:**

The adapted SNSME questionnaire in the current setting is valid and the use of social media for learning is influenced by gender and the learning environment. This study highlights the importance of supporting students in using social media for learning purposes as well as using social media as a means to increase their SRL skills.

## Introduction

Social media is widely used by medical students, including for learning purposes. It allows them to be involved in the communities of inquiry where they can share, express, and engage in the development of knowledge [1, 2]. Different social media platforms offer diverse methods of communication and collaboration opportunities, and consequently they can be useful for learning both formally and informally [3]. Guraya et al. developed an instrument to assess the use of social networking sites (SNS) in medical education with 20 questions. Their study showed that in two universities in Saudi Arabia, a small proportion of students (37%) utilized social media for learning purposes [4]. However, following the COVID-19 pandemic, the use of social media platforms such as YouTube, WhatsApp, and Blackboard to support learning or teaching purposes has increased among undergraduate medical students and teachers [5]. Male students tend to use social media for exchanging information and more senior students utilize social media for learning to a greater extent compared to their junior counterparts [4].

The use of social media by medical students requires an extent of self-regulation [6]. It has been suggested that social media can help students apply their self-regulated learning (SRL) processes through socially mediated knowledge and networked learning, particularly in setting learning goals, managing information strategically, and self-monitoring their progress [6]. Using social media for learning encourages students to evaluate information, exchange knowledge, and manage distractions; therefore, SRL skills in goal setting, environmental structuring, performance control, and self-evaluation are required [7]. This can be challenging when students use social media voluntarily not necessarily driven by their course learning outcomes and assessments [7, 8].

The SRL concept underlines students’ abilities to actively govern, observe, and adjust their learning processes to achieve the learning outcomes [9], and is therefore highly relevant to this study. SRL consists of three cyclical dynamic phases: (1) Preparation and learning outcome definition, (2) Implementation of learning activities, and (3) Reflection [10]. Existing studies exploring the relationships between the use of social media and SRL suggest inconsistent results; for example, one study shows that increased use of Facebook correlates with the deterioration of academic performance. On the other hand, another study highlights that students who are active in using social media also show increased academic performance [11]. From the SRL perspective, social media can actually encourage goal-making, self-monitoring, motivation, and task strategies. However, there can also be a negative effect on self-evaluation and time management [6], which should underscore the importance of self-awareness and self-control in using social media appropriately.

In relevance with the SRL, social media enables the development of a personal learning environment (PLE) [6] in which students become part of the communities of inquiry that have the potential to support their learning [12]. PLEs enable adult learners to ‘create, organize, and share content’ and become independent, self-regulated learners [13]. Such networks and environments can be directed individually to pursue educational goals, and are beyond organizational and institutional boundaries [6, 12, 13].

Studies exploring the use of social media tend to elaborate on the information and knowledge that can be shared through different platforms rather than focusing on how students can navigate the use of social media by incorporating their SRL skills. Nonetheless, a study by Zhou et al. [7] has explored the use of SRL in navigating social media for voluntary learning among university students in Singapore. The importance of identifying social media use for learning purposes and its relationships with SRL skills among medical students due to the need for developing future adaptive practitioners with increased use of social media [14] is a key motivation for the present study. In addition, it is suggested that male and female medical students use social media for their learning differently [4], which might be related to their different learning needs [15]. Considering the undergraduate medical program context within this study [16] with 3.5-4-year preclinical and 2-year clinical programs, as well as the role of public and private medical schools, our intention is to assess whether these factors are related to the use of social media for learning and the SRL skills.

For the purposes of this study, we selected the Social Networking Sites for Medical Education (SNSME) questionnaire developed by Guraya et al. [4] to identify the use of social media for learning among medical students, and the Indonesian-validated Motivated Strategies for Learning Questionnaire (MSLQ) to describe the students’ SRL skills. Furthermore, the study is structured around three research questions: (a) Is the SNSME questionnaire valid for use in Indonesian settings? (b) Are there any differences between the SNSME and MSLQ scores of male and female students, preclinical and clinical year medical students, and students from private and public medical schools? (c) What is the relationship between medical students’ SRL skills (as measured by MSLQ) and their use of social media for learning (as measured by the SNSME questionnaire)?

## Methods

### Context

The study was conducted among medical students from private and public medical schools in Indonesia. Indonesia is an archipelago with a population of around 270 million and 93 medical schools, the majority of which are private rather than public. All medical schools implement competency-based medical curricula which consider expected national standards of learning outcomes or graduate competence. Blended and hybrid learning methods have been introduced widely, strengthening during the pandemic. As of January 2023, a total of 167 million Indonesians (60.4% of the total population) use social media [17]; the top five most used social media platforms are WhatsApp, Instagram, Facebook, TikTok and Telegram [18]. Despite this widespread use of social media in our context, the current uses in terms of students’ learning processes in different medical schools have not been recognized.

### Design

This was a cross-sectional study regarding the use of social media for educational purposes and its relationship with students’ SRL skills using validated questionnaires.

### Participants

The respondents were medical students residing in Indonesia who have been using social media for their learning. The population of the study comprised preclinical and clinical medical students from 43 out of 93 medical schools (public and private) in Indonesia. We invited medical educators and/or medical student representatives from the 43 medical schools across Indonesia to collaborate in this study and facilitate the data collection within their settings. This approach provided access to 45,578 medical students at both preclinical and clinical stages in those medical schools. Based on the total items of the instruments used in this study (81 MSLQ items and 19 SNSME items) and the aim of validating the questionnaire quantitatively, especially for SNSME, a minimum of 500 respondents were sought [19].

### Instruments

Two questionnaires were used: (1) the MSLQ, which has already been validated for use in Indonesian settings, to measure students’ SRL skills [20, 21]; and (2) the SNSME questionnaire [4]. The SNSME questionnaire originally consists of 20 items and is completed using a Likert scale from 1 (never) to 5 (every day) for items 1–6 and from 1 (strongly disagree) to 5 (strongly agree) for the remaining items. The MLSQ consists of 81 items with a Likert scale ranging from 1 (not at all true of me) to 7 (very true of me). The authors were aware that the MSLQ items focus on students’ learning within the confines of one formal course. In this study, we asked students from different medical schools to complete the items by reflecting on the current course that they were studying when they filled in the questionnaires. Considering the different curricula used in Indonesian medical schools, we anticipated that students would reflect on various courses at the time of the study; this provided an opportunity to assess students’ SRL skills with a more general perspective rather than focusing on one specific course.

The SNSME questionnaire was translated into Indonesian and back-translated into English by two different teams: AF & CH, who have good English proficiency and understanding of the content, translated the SNSME questionnaire from English to Indonesian, and NG & EW back-translated the questionnaire from Indonesian to English without reference to the original questionnaire. The authors discussed the comparability of both versions. Before completing the SNSME questionnaire, students were asked to identify their use of social media by listing their most frequently used platforms, such as Instagram, Facebook, WhatsApp, YouTube, TikTok, Twitter, LinkedIn, etc. The different uses of these social media platforms are reported in another publication [22].

A pilot study involving 20 preclinical and clinical year medical students from two medical schools was conducted using both questionnaires. Some inputs were recorded regarding clarity in the questions on social media; since different types of social media can be used for different purposes, it is necessary to provide definitions of the use of social media for learning. The time required to complete both questionnaires was also noted. Inputs from the pilot study were used to revise the SNSME items where appropriate and the study was also adjusted so that participants would be able to complete the questionnaire in several attempts rather than a single session in the Google form.

### Data collection

The authors approached different medical schools in Indonesia through medical teachers or student representatives to collect the data. Consecutive sampling was conducted. Formal permissions from the medical schools’ authorities were also sought. The medical educators and/or medical student representatives reached out to the students directly via their internal communication systems. Following access to medical students, the surveys were distributed via a Google form. Agreement to participate in the survey was included in the form and completion of the questionnaire also signaled consent from the respondents. The students completed the Indonesian version of the questionnaires.

### Data analysis

Collected data were subjected to cleaning, and further data analysis was carried out. The data analysis was completed using IBM Statistical Package for Social Science version 28. Exploratory Factor Analysis (EFA) with Principal Component Analysis and Varimax Rotation was completed for the SNSME questionnaire to support its construct validity within the current study. Reliability analysis using Cronbach’s alpha was completed for data from both questionnaires to assess their internal consistencies. Finally, descriptive statistics elaborating on the scores of the scales and subscales for both questionnaires, the comparison of scores based on characteristics under study, and correlation analysis were also completed. These analyses considered the normal or abnormal distribution of the data.

The study has been approved by the Faculty of Medicine Universitas Indonesia ethical committee (No: KET-44/UN2.F1/ETIK/PPM.00.02/2022).

## Results

A total of 1,122 medical students participated in this study, which fulfills the minimum sample we aimed for this study. Of those, 387 (34.5%) were male and 785 (65.5%) were female; 628 (56%) were preclinical year students and 494 (44%) were clinical year students. A total of 272 students (24.2%) were from public medical schools and 850 (75.8%) were from private medical schools. These characteristics reflect the current distribution of medical students in the country.

### Exploratory factor analysis and reliability analysis of the SNSME questionnaire (*N* = 1122)

**Fig. 1:**
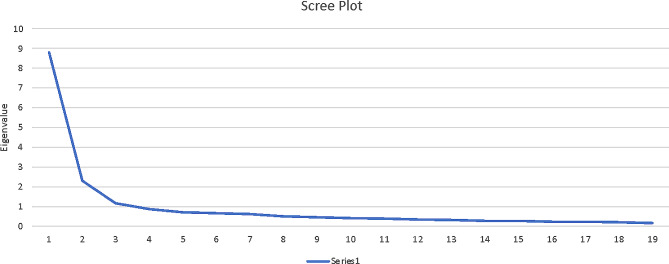
SNSME scree plot

The results showed adequate data for the application of EFA (KMO 0.940, the Bartlett test of sphericity is significant (Chi-square 14.117, df = 190, *p* = 0.000)). Each item shows commonalities with other items with a minimum correlation of 0.3, and each item has a correlation with at least one item in the questionnaire except for item 20 – ‘I believe that social networking sites are inappropriate for sharing classroom materials’, which shows correlation below 0.3 with all other items. Given this, we decided to remove this item from the questionnaire and further analysis. Tabachnick and Fidell recommended inspecting the correlation matrix for correlation coefficients over 0.3 for factorability of the dataset [23]. The factorability of the dataset means that when the items have similar underlying dimension(s), we would expect the items to correlate with each other. Therefore, any items with a lot of correlations below 0.3 with other items (such as item 20 in this study) can be excluded for further steps of analysis [24, p 648]. With the 19 items fulfilling the criteria for factorability, we reran the EFA with 19 items (SNSME 1–19). Based on the eigenvalue > 1, scree plot and parallel analysis [25], three components/subscales were extracted covering 64.5% of the variance (Table 1; Fig. 1; Table 2).

**Table 1:** SNSME component extraction

Component		Initial Eigenvalues	Extraction Sums of Squared Loadings						
		% of Variance	Cumulative %	Total	% of Variance	Cumulative %			
**1**	**8.783**		**46.226**	**46.226**	**8.783**	**46.226**	**46.226**		
**2**	**2.308**		**12.147**	**58.373**	**2.308**	**12.147**	**58.373**		
**3**	**1.168**		**6.146**	**64.520**	**1.168**	**6.146**	**64.520**		
4	0.867		4.564	69.083					
5	0.719		3.783	72.866					
6	0.684		3.599	76.465					
7	0.627		3.301	79.767					
8	0.507		2.666	82.433					
9	0.446		2.350	84.783					
10	0.421		2.216	86.999					
11	0.396		2.085	89.083					
12	0.350		1.840	90.924					
13	0.314		1.653	92.576					
14	0.301		1.586	94.162					
15	0.283		1.488	95.650					
16	0.235		1.235	96.884					
17	0.226		1.188	98.072					
18	0.201		1.057	99.129					
19	0.165		0.871	100.000					

Extraction Method: Principal Component Analysis. The bold values are those with Eigenvalue more than 1

**Table 2:** Parallel analysis [26]

Component/factor	Eigenvalue from the dataset	Eigenvalue from the parallel analysis	Interpretation
1	**8.783**	**1.223383**	Factor is retained
2	**2.308**	**1.183835**	Factor is retained
3	**1.168**	**1.153150**	Factor is retained
4	0.867	1.126333	Factor is not retained
5	0.719	1.102416	Factor is not retained

The bolds values signify the comparison of Eigenvalue from data set and parallel analysis

Based on principal component analysis and further varimax rotation, all SNMSE items were grouped into the three subscales. The subscales were considered stable as each consisted of a minimum of three items and there were no cross-loadings (Table 3).

**Table 3:** Rotated matrix of the SNSME questionnaire with 19 items and 3 subscales

Items		Components		
		1	2	3
SNSME_1	Seberapa sering Anda menggunakan email untuk berbagi informasi yang berkaitan dengan proses pendidikan Anda? *How often do you use email for sharing information for educational purposes?*	0.005	0.491	0.391
SNSME_2	Seberapa sering Anda menggunakan situs jejaring sosial (seperti Instagram, Facebook, YouTube, Twitter, LinkedIn, Line, WhatsApp and TikTok) untuk dapat tetap terhubung dengan teman dan dosen Anda? *How often do you use social networking sites (e.g., Instagram, Facebook, YouTube, Twitter, LinkedIn, Line, WhatsApp and TikTok) to keep in touch with peers and tutors?*	0.314	0.803	− 0.017
SNSME_3	Seberapa sering Anda menggunakan situs jejaring sosial (seperti Instagram, Facebook, YouTube, Twitter, LinkedIn, Line, WhatsApp and TikTok) untuk berbagi informasi yang berkaitan dengan pendidikan Anda? *How often do you use social networking sites (e.g., Instagram, Facebook, YouTube, Twitter, LinkedIn, Line, WhatsApp and TikTok) to share education-related information?*	0.231	0.791	0.231
SNSME_4	Seberapa sering Anda menggunakan situs jejaring sosial untuk berbagi informasi mengenai penelitian dan inovasi di bidang kedokteran? *How often do you use social networking sites for sharing research or innovations in the medical field?*	0.158	0.491	0.598
SNSME_5	Seberapa sering Anda membaca blog atau Wikis mengenai informasi yang berkaitan dengan pendidikan? *How often do you read blogs or Wikis for education-related information?*	0.140	0.167	0.737
SNSME_6	Seberapa sering Anda memberikan kontribusi pada blog atau Wikis untuk berbagi informasi atau mendiseminasikan pengetahuan? *How often do you contribute to blogs or Wikis to share information, or disseminate knowledge?*	− 0.062	0.013	0.864
SNSME_7	Situs jejaring sosial membantu saya dalam mengumpulkan materi pembelajaran *Social networking sites help me in collection of educational materials*	0.766	0.252	0.065
SNSME_8	Situs jejaring sosial bermanfaat dalam pembelajaran secara kolaborasi dan pembelajaran antar-teman sebaya (peer-to-peer learning) *Social networking sites are helpful in collaborative and peer-to-peer learning*	0.793	0.243	0.023
SNSME_9	Situs jejaring sosial berguna dalam mengembangkan kemampuan membaca dan menulis dalam situs web *Social networking sites are useful in developing reading and writing web skills*	0.693	0.038	0.302
SNSME_10	Situs jejaring sosial menyediakan kesempatan untuk bertemu secara virtual dengan peserta didik lain atau dosen *Social networking sites provide opportunity for virtual meetings with other students and tutors*	0.801	0.189	0.027
SNSME_11	Situs jejaring sosial membantu saya untuk berkomunikasi dengan teman mengenai tugas atau proyek yang sedang dikerjakan *Social networking sites help me to communicate with peers about class projects*	0.824	0.235	− 0.119
SNSME_12	Situs jejaring sosial membantu saya untuk mengakses sumber pembelajaran *Social networking sites help me to access educational resources*	0.827	0.260	− 0.071
SNSME_13	Situs jejaring sosial membantu saya untuk mendapatkan referensi untuk penelitian *Social networking sites help me to retrieve educational references for research*	0.780	0.215	0.101
SNSME_14	Situs jejaring sosial memfasilitasi pengembangan profesional saya terkait dengan kemampuan teknologi *Social networking sites facilitate my professional development of technological skills*	0.807	0.115	0.116
SNSME_15	Situs jejaring sosial bermanfaat dalam proses komunikasi dengan teman sekelas mengenai topik pembelajaran dalam modul atau mata kuliah *Social networking sites are useful in communicating with classmates about course-related topics*	0.846	0.205	− 0.035
SNSME_16	Saya merasa bahwa situs jejaring sosial bermanfaat saat menjelang ujian, karena saya bisa mendapatkan jawaban atau penjelasan langsung dari teman sebaya, tanpa harus mencari jawabannya melalui buku *I have found social networking sites useful during the pre-exam period when I get an instant answer/explanation from my peers, instead of going through the books*	0.567	− 0.111	0.137
SNSME_17	Saya merasa bahwa situs jejaring sosial bermanfaat untuk berbagi catatan atau materi kuliah *I have found social networking sites useful for sharing notes and lectures*	0.810	0.116	0.001
SNSME_18	Saya merasa bahwa situs jejaring sosial bermanfaat untuk kepentingan pendidikan *I have found social networking sites useful for educational purposes*	0.850	0.157	− 0.008
SNSME_19	Peserta didik membutuhkan supervisi dan bimbingan dalam menggunakan situs jejaring sosial untuk kepentingan pendidikan secara tepat *Students need supervision and guidance for the appropriate use of social networking sites for educational purposes*	0.624	− 0.003	0.246

Extraction Method: Principal Component Analysis

Rotation Method: Varimax with Kaiser Normalization

a. Rotation converged in 5 iterations

Those subscales were interpreted as follows:

**Subscale 1 (SNSME 7–19; 13 items).** Attitudes towards the use of social media for learning and knowledge development (13 items).

**Subscale 2 (SNSME 1–3, 3 items).** The use of social media for information sharing and interaction (3 items).

**Subscale 3 (SNSME 4–6, 3 items).** The use of social media for knowledge development and research (3 items).

The reliability of each SNSME subscale was excellent, and the Cronbach’s alpha for the SNSME questionnaire as a whole (items 1–19) was 0.906. The internal consistency scores for subscales 1 and 3 were 0.941 and 0.707, respectively, and the internal consistency of subscale 2 was slightly lower (with a Cronbach’s alpha of 0.651).

The total score for each subscale was also calculated. A higher score reflects more frequent or consistent use of social media and a more positive attitude towards the use of social media for learning [4]. Upon evaluation of any data which were not normally distributed, the median and range scores were reported. The SNSME scores based on gender, education stage, and type of medical school (private or public) were also described for each subscale (Table 4). There were significant differences between the total SNSME score, subscale 2, and subscale 3 based on respondents’ gender with the highest scores for female participants (80 vs. 82, p 0.007; 12 vs. 13, p 0.002; and 9 vs. 10, p 0.004, respectively). The SNSME score did not show significant differences between preclinical and clinical students. Students from public medical schools presented higher scores for the SNSME total and subscale 1 (83 vs. 81, p 0.007; and 61 vs. 59, *p* < 0.001, respectively).

**Table 4:** SNSME scores based on gender and education stage

SNSME scores	Male (*n* = 387)		Female (*n* = 735)		p	Preclinical (*n* = 628)		Clinical (*n* = 494)		p	Public (*n* = 272)		Private (*n* = 850)		p
	Median	Range	Median	Range		Median	Range	Median	Range		Median	Range	Median	Range	
Total score	**80**	29–95	**82**	27–95	0.007*	82	27–95	81	29–95	0.23	**83**	52–95	**81**	27–95	0.007*
Subscale 1 – Attitudes toward the use of social media for learning and knowledge development (13 items)	59	13–65	60	13–65	0.258	60	13–65	59	14–65	0.29	**61**	39–65	**59**	13–65	< 0.001*
Subscale 2 – The use of social media for information sharing and interaction (3 items)	**12**	4–15	**13**	5–15’	0.002*	13	5–15	13	4–15	0.66	13	6–15	13	4–15	0.653
Subscale 3 – The use of social media for knowledge development and research (3 items)	**9**	3–15	**10**	4–15	0.004*	10	3–15	10	3–15	0.64	10	3–15	10	3–15	0.273

*statistically significant. Other bold values highlight the median of the sub-scales with significant difference

### MSLQ reliability analysis (*N* = 1122)

The reliability analysis of the MSLQ was also excellent. The total MSLQ score had a Cronbach’s alpha of 0.973. The motivation scales (31 items) showed a reliability of 0.960, with a range of 0.774–0.928 for the value, expectancy, and affective components. The learning strategies scales (50 items) also had excellent internal consistency (Cronbach’s alpha = 0.968) with a range of 0.854–0.922 for their components and subcomponents (cognitive and metacognitive strategies (rehearsal, elaboration, organization, critical thinking, and metacognitive self-regulation) and resource management strategies (time and study environment, effort regulation, peer learning, and help-seeking)). For the total of 81 items, the median MSLQ score in this study was 422 (range 136–549); the motivation subscales had a score of 172 (range 36–217), and the learning strategies subscales had a score of 254 (range 100–340). The total MSLQ scores of students from preclinical and clinical years as well as public and private medical schools presented significant differences (426 vs. 418, p 0.003; and 436 vs. 418, *p* < 0.001, respectively). Table 5 further describes the MSLQ scores based on gender, education stage, and medical school type.

**Table 5:** MSLQ scores based on gender, education stage, and medical school type

MSLQ scores	Male (*n* = 387)		Female (*n* = 735)		p	Preclinical stage (*n* = 628)		Clinical stage (*n* = 494)		p	Public (*n* = 272)		Private (*n* = 850)		p
	Me dian	Range	Me dian	Range		Me dian	Range	Me dian	Range		Me dian	Range	Me dian	Range	
Total MSLQ (81 items)	420	136–534	423	194–549	0.657	**426**	136–536	**418**	194–549	0.003*	**436**	221–519	**418**	136–549	< 0.001*
MSLQ A Motivation scales (31 items)	170.5	36–217	173	54–217	0.215	**175**	36–217	**168**	61–217	< 0.001*	175	74–217	171	36–217	0.084
MSLQ B Learning strategies scales (50 items)	253	100–332	254	115–340	0.511	**254**	100–332	**253**	115–340	0.224	**261**	120–326	**250**	100–340	0.004*
MSLQ A1 Motivation scales – value component (14 items)	80	16–98	81	19–98	0.155	**83**	16–98	**78**	28–98	< 0.001*	**82**	34–98	**80**	16–98	0.026*
Intrinsic goal orientation (4 items)	22	4–28	21	6–28	0.213	**22**	4–28	**20.5**	7–28	< 0.001*	22	7–28	21	4–28	0.123
Extrinsic goal orientation (4 items)	24	4–28	24	6–28	0.111	**25**	4–28	**23**	7–28	< 0.001*	**24**	**8**–**28**	**24**	**4**–**28**	0.012*
Task value (6 items)	41	8–49	41	7–49	0.133	**42**	7–49	**40**	14–49	< 0.001*	**42**	18–49	**41**	7–49	< 0.001*
MSLQ A2 Motivation scales – expectancy component (12 items)	67.5	15–84	67	24–84	0.179	**68**	15–84	**65**	24–84	< 0.001*	**69**	25–84	**66**	15–84	0.026*
Control of learning beliefs (4 items)	23	5–28	24	4–28	0.31	**24**	4–28	**23**	8–28	< 0.001*	**24.5**	8–28	**23**	4–28	< 0.001*
Self-efficacy for learning and performance (8 items)	44	10–56	43	15–56	0.154	**42**	15–56	**44**	10–56	0.002*	**44**	17–56	**43**	10–56	0.046*
MSLQ A3 Motivation scales – affective component (5 items)	23	5–35	24	5–35	0.201	**24**	5–35	**23**	8–35	< 0.001*	24	8–35	23	5–35	0.740
Test anxiety (5 items)	23	5–35	24	5–35	0.201	**24**	5–35	**23**	8–35	< 0.001*	24	8–35	23	5–35	0.740
MSLQ B1 Learning strategies scales – cognitive and metacognitive strategies (31 items)	161	45–213	162	60–214	0.755	163.5	45–214	161	60–214	0.073	**169.5**	62–213	**159**	45–214	< 0.001*
Rehearsal (4 items)	22	4–28	22	6–28	0.386	22	4–28	22	6–28	0.062	**23**	8–28	**22**	4–28	0.004*
Elaboration (6 items)	32	6–42	33	11–42	0.887	32	6–42	32	11–42	0.389	**35**	11–42	**32**	6–42	< 0.001*
Organization (4 items)	21	4–28	21	8–28	0.704	21	4–28	21	7–28	0.396	**22.5**	7–28	**20**	4–28	< 0.001*
Critical thinking (5 items)	26	7–35	25	8–35	0.517	26	7–35	26	8–35	0.408	**27**	10–35	**25**	7–35	< 0.001*
Metacognitive self-regulation (12 items)	61	18–80	61	27–84	0.752	62	18–84	61	27–84	0.053	**64**	27–80	**61**	18–84	< 0.001*
MSLQ B2 Learning strategies scales - cognitive and metacognitive strategies - Resource management strategies (19 items)	89	47–127	90	47–127	0.171	90	47–127	89	55–127	0.315	**91**	55–125	**89**	47–127	0.005*
Time and study environment (8 items)	**36**	21–56	**37**	21–56	0.028*	**37**	21–56	**36**	23–56	0.035*	37	21–56	36	21–56	0.250
Effort regulation (4 items)	**17**	8–28	**18**	9–28	0.017*	18	10–28	17	8–28	0.252	18	9–28	17	8–28	0.294
Peer learning (3 items)	15	3–21	15	3–21	0.958	15	3–21	15	4–21	0.23	**16**	5–21	**15**	3–21	0.025*
Help-seeking (4 items)	19	5–28	18	6–28	0.432	18	5–28	18	6–27	0.999	19	6–26	18	5–28	0.052

*statistically significant. Bold values highlight the median of the sub-scales with significant difference

### SNSME and MSLQ correlations

Finally, Table 6 demonstrates the correlations between the SNSME questionnaire and MSLQ in this study. All scales and subscales show statistically significant low to moderate correlations (R 0.195–0.462), except for the MSLQ motivation scales– affective component with the SNME subscale 3 score.

**Table 6:** SNSME and MSLQ correlations

MSLQ scores	SNME scores							
	Total score		Subscale 1 score		Subscale 2 score		Subscale 3 score	
	R	p	R	p	R	p	R	p
MSLQ total score (81 items)	0.448	< 0.001	0.448	< 0.001	0.334	< 0.001	0.252	< 0.001
MSLQ A Motivation scales (31 items)	0.458	< 0.001	0.438	< 0.001	0.302	< 0.001	0.202	< 0.001
MSLQ B Learning strategies scales (50 items)	0.454	< 0.001	0.408	< 0.001	0.316	< 0.001	0.252	< 0.001
MSLQ A1 Motivation scales – value component (14 items)	0.462	< 0.001	0.442	< 0.001	0.302	< 0.001	0.204	< 0.001
MSLQ A1 Motivation scales – expectancy component (12 items)	0.45	< 0.001	0.42	< 0.001	0.301	< 0.001	0.227	< 0.001
MSLQ A1 Motivation scales – affective component (5 items)	0.207	< 0.001	0.222	< 0.001	0.137	< 0.001	0.041	0.175
MSLQ B1 Learning strategies scales – cognitive and metacognitive strategies (31 items)	0.459	< 0.001	0.414	< 0.001	0.312	< 0.001	0.26	< 0.001
MSLQ B2 Learning strategies scales – cognitive and metacognitive strategies – Resource management strategies (19 items)	0.39	< 0.001	0.348	< 0.001	0.293	< 0.001	0.195	< 0.001

## Discussion

This study involved medical students in different schools in Indonesia and followed a robust process of questionnaire adaptation and EFA. The SNSME questionnaire’s validity for use in the current setting has been highlighted and consists of three subscales: (1) attitudes towards the use of social media for learning and knowledge development, (2) the use of social media for information sharing and interaction, and (3) the use of social media for knowledge development and research. Compared to female students, male students had lower scores with regard to SNSME subscales 2 and 3, students from public medical schools showed higher scores than their private school counterparts for SNSME subscale 1, and there was no difference in terms of SNSME scores between preclinical and clinical year students. On the other hand, there were significant differences in the total MSLQ scores of preclinical and clinical year students as well as public and private medical schools. There were low to moderate correlations between the total and subscale scores for the SNSME questionnaire and the MSLQ.

While the use of social media does provide opportunities for students to develop their community of inquiry [1, 2], this study shows that, overall, students had high scores in terms of utilizing social media for learning purposes as measured by the SNSME questionnaire, which was first developed by Guraya et al. [4] to identify students’ perceptions regarding the use of social media for learning. The study has reported the results for the 19 items and highlights that students frequently use social media to stay in touch with their peers and tutors, often using social media to share education-related information such as notes and lectures [4]. There was no further elaboration on the SNSME questionnaire as a scale, as suggested in the present study. Studies conducted during and after the COVID-19 pandemic showed the increased need for connectivity among learners, teachers, and communities of practice in acquiring new knowledge and skills [5, 14], including issues not usually discussed in the formal medical curricula [14]; this may explain the high scores recorded in this study.

In addition, this study shows that female students tend to use social media more frequently for information sharing, interaction, knowledge development, and research, and also have more positive attitudes towards the use of social media for learning compared to their male counterparts. This present study also partly confirms a study by Guraya et al. [4] where female students scored significantly higher for SNSME items 1–5, which reflects the frequency of use of social media for different aspects of learning. On the other hand, in contrast to the current research, male students in the study by Guraya et al. showed more positive attitudes compared to female students, which may suggest that male students consider social media to be a reliable and convenient source for learning [27]. Social interactions through media differ between genders [28] and gender does influence media use [29]. This underlines the different ways in which social media is used with regard to learning, likely driven by the different learning needs of students and their aims in using social media; for example, male students are more likely to use social media for entertainment and to develop networks rather than for information gathering and learning purposes [30–32].

In this study, students from private medical schools showed slightly lower scores compared to those from public medical schools for the SNSME total and subscale 1. This suggests that private school students’ attitudes towards the use of social media for learning were rather less positive compared to public school students. This might be explained by different factors including the recognition of social media as a learning resource among teachers [11], the facilitation of the use of social media within the educational environment [33], and the formal acknowledgment of the benefits of using social media as a learning resource, for instance, in increasing accessibility and flexibility, as well as risks including distraction and technical difficulties [34]. Considering the possible differences in the learning environment, attitudes towards the use of social media as a learning resource in public and private medical schools could also be influenced by the overall curriculum implementation, interactions between students and between teachers and students, and available learning facilities [34]. Future qualitative studies should be conducted to explore this issue further.

The SRL scores of students participating in this study demonstrated that preclinical year students had higher scores compared to clinical year students; moreover, students from public medical schools showed higher SRL scores, especially with regard to learning strategies. Since social media for learning is underpinned by learning theories based on constructivism and connectivism [35, 36] which allow students to create their own knowledge and facilitate their engagement, self-reflection, and active learning [37], the SRL of medical students is a key aspect of utilizing social media for learning. On the other hand, social media may also influence SRL skills. While it is evident that the primary use of social media by medical students is not for learning, it may help with their SRL skills, i.e., setting personal goals, managing information, and engaging in monitoring and evaluating their learning [6]. In addition, self-regulated social media use in a voluntary and informal manner enables students to differentiate useful information and misinformation as well as to critically reflect on the blurred boundary between learning activities and social activities [7]. Therefore, the rather lower MSLQ scores of clinical year students and students from private schools might call for further support for the students so that they have the capabilities to utilize social media for their learning. In addition, given the inevitable urge to become lucrative and commercial, it can be challenging for private medical schools to maintain their quality of education and student support [38]. The difference in the levels of SRL in public and private medical schools might also be explained by the different student selection mechanisms within these settings; however, this issue has not been investigated in this paper and further study is necessary.

The results of this study showed a low to moderate correlation between social media usage and students’ SRL skills, except for the affective components of motivation scales as the subscale assessed levels of anxiety in facing examinations, which were not directly related to social media use. The correlation between social media usage and SRL in this study demonstrates how social media can enhance the student experience in terms of collaborative learning, which increases their SRL ability. However, the low to moderate correlation between social media usage and SRL may be caused by the minimal use of social media as a learning platform compared to its usage for entertainment or other purposes. Therefore, medical education institutions may need to formally and explicitly use social media as a learning modality, especially to facilitate collaborative learning among students [35]. Indeed, a significant correlation between using social media as a learning modality and medical students’ academic performance has been demonstrated [39]. In addition, instructional designs in the curriculum allowing the use of social media in a progressive manner which can support SRL development would also be beneficial [6].

This study has several implications. First, the use of social media for learning and SRL skills should be supported concurrently in undergraduate medical education, allowing students to benefit from creating their learning spaces through social media while developing their SRL skills. Second, since social media use has become so closely interwoven into medical students’ lives, addressing and enhancing their SRL skills should be part of the current curriculum. Third, faculty may consider identifying students’ SRL skills during each stage of their education as part of a formative assessment; as part of this process, contributing factors besides the use of social media should also be identified. Fourth, in accordance with the need to address social media use and support students’ SRL skills in the curriculum, appropriate faculty development programs should be established.

The authors are also aware of the study’s limitations. First, the nature of the cross-sectional design does not allow an analysis of the causal relationship between the attitudes toward social media for learning and the students’ SRL skills. A more longitudinal study which enables an adequate follow-up is warranted. Second, the current quantitative data provide important profiles necessary to explore the use of social media for learning and students’ SRL skills; however, this study did not explore the reasons behind those relationships. Consequently, a follow-up employing a qualitative research design can be conducted in the future. Third, this study was completed in a single country and therefore the generalizability of the results may be an issue. However, since the study involved a large sample from several different medical schools (1122 samples from 43 medical schools) in this setting, we still hope to highlight the importance of further efforts to utilize social media as part of students’ learning processes while supporting their SRL skills.

## Conclusion

This study shows that the adapted SNSME questionnaire in the current setting is valid and that the use of social media for learning can be influenced by both the gender and origin of medical students, signifying the various learning needs, the role of the learning environment, and the curriculum. Preclinical year students and students from public medical schools have a higher competence with regards to SRL compared to those studying in their clinical year and from private medical schools, respectively. Finally, the use of social media is minimally or moderately correlated with the students’ SRL skills. Therefore, support should be provided for students’ SRL skills in using social media for learning purposes, and the use of social media as a means to increase those skills should be encouraged.

## Data Availability

The datasets generated and/or analyzed during the current study are not publicly available due to conditions of participants’ consent but are available from the corresponding author on reasonable request.
